# The novel tool of cell reprogramming for applications in molecular medicine

**DOI:** 10.1007/s00109-017-1550-4

**Published:** 2017-06-08

**Authors:** Moritz Mall, Marius Wernig

**Affiliations:** Department of Pathology and Institute for Stem Cell Biology and Regenerative Medicine, Stanford University School of Medicine, Stanford, CA 94305 USA

**Keywords:** Cell fate, Reprogramming, Stem cell biology

## Abstract

Recent discoveries in the field of stem cell biology have enabled scientists to “reprogram” cells from one type to another. For example, it is now possible to place adult skin or blood cells in a dish and convert them into neurons, liver, or heart cells. It is also possible to literally “rejuvenate” adult cells by reprogramming them into embryonic-like stem cells, which in turn can be differentiated into every tissue and cell type of the human body. Our ability to reprogram cell types has four main implications for medicine: (1) scientists can now take skin or blood cells from patients and convert them to other cells to study disease processes. This disease modeling approach has the advantage over animal models because it is directly based on human patient cells. (2) Reprogramming could also be used as a “clinical trial in a dish” to evaluate the general efficacy and safety of newly developed drugs on human patient cells before they would be tested in animal models or people. (3) In addition, many drugs have deleterious side effects like heart arrhythmias in only a small and unpredictable subpopulation of patients. Reprogramming could facilitate precision medicine by testing the safety of already approved drugs first on reprogrammed patient cells in a personalized manner prior to administration. For example, drugs known to sometimes cause arrhythmias could be first tested on reprogrammed heart cells from individual patients. (4) Finally, reprogramming allows the generation of new tissues that could be grafted therapeutically to regenerate lost or damaged cells.

## Introduction

The fate of a cell is an integral of its morphological and functional makeup that is in turn dictated by its transcriptional, epigenetic, proteomic, and metabolic configuration. Cellular fate is changing during development as the multicellular organism develops from a single totipotent cell to yield billions of specialized cells that make up the human body. Ever since Hans Spemann showed in 1923 that the blastomeres of a 16-cell salamander embryo are all equivalent to the totipotent zygote, it remained an open question whether more differentiated cells irreversibly lose this developmental potential [1]. It was debated whether perhaps even genetic material might be lost during differentiation, which would eliminate the totipotent potential of specialized cells.

**Fig. 1:**
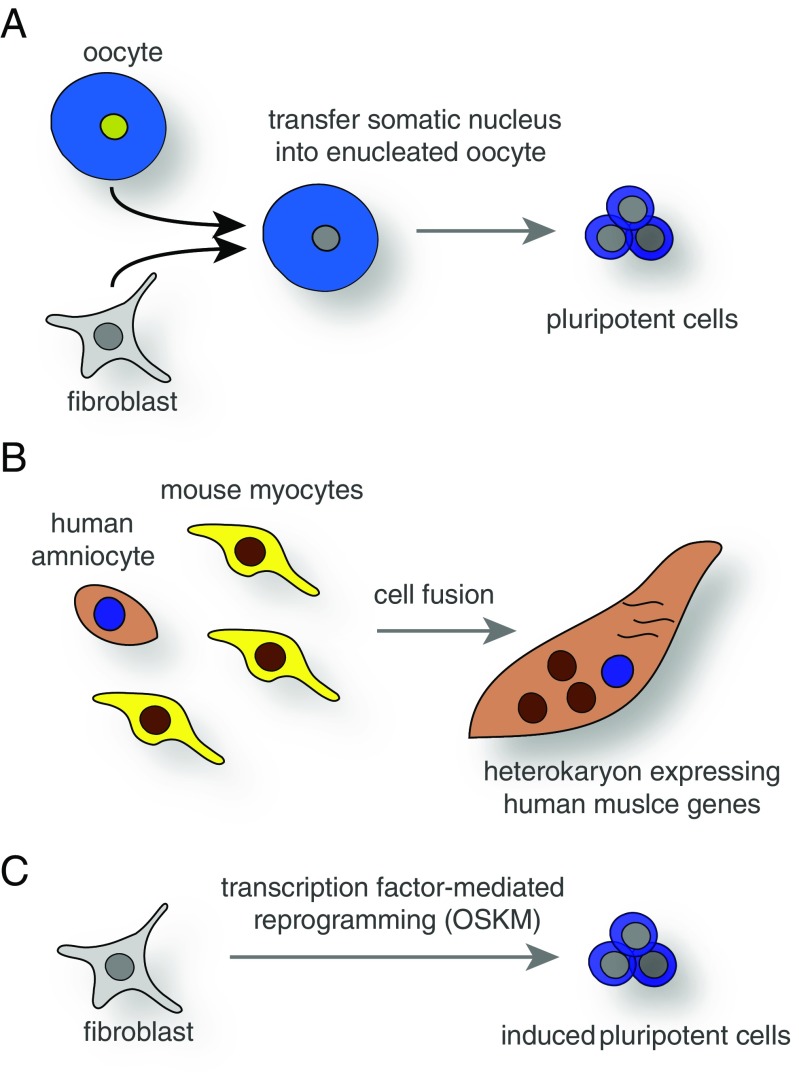
Common technologies to reprogram cell fate. **a** Somatic cell nuclear transfer (SCNT), in which an oocyte is enucleated to receive a nucleus from a donor cell such as a fibroblast uses the cytoplasmic machinery to reprogram the donor cell to pluripotency. Similar methods were used to clone entire animals such as Dolly the sheep and generate human stem cell lines. **b** Analogous to SCNT diffusible factors can reprogram the expression program of a donor cell such as a human amniocyte upon induced cell fusion with heterologous cells such as mouse myocytes to induce the expression of human muscle genes. **c** Alternatively, strong cell fate determination transcription factors can be overexpressed using different methods to change a cell fate. For example, the transcription factors Oct4, Sox2, Klf4, and c-Myc (OSKM) can convert a fibroblast into an induced pluripotent stem cell

One of the first decisive experiments was the nuclear transfer of specialized cell nuclei into oocytes (Fig. 1a). These experiments first done in frogs showed that specialized cells can be reprogrammed to totipotency and can give rise to a new animal [2, 3]. Thus, even specialized cells can activate the entire program of embryonic development. In addition, adult cells can adapt and change quite dramatically upon certain environmental conditions. For example, the respiratory epithelium in the lungs of smokers can convert into squamous cells, and the esophagus epithelium can adopt the morphology of gastric epithelium in a process called metaplasia [4]. But also in hematopoietic tumors, cells have been found to transdifferentiate from one blood lineage to another [5, 6]. There is also evidence that pancreatic α or δ cells can change to β cells upon injury [7, 8]. An additional example for induced lineage plasticity was provided by cell fusion experiments (Fig. 1b) [9, 10].

More recently, specific transcription factors or combinations thereof have been identified to induce such lineage conversions (Fig. 1c). Among others, MyoD was found to induce muscle fates in fibroblasts [11], Pax6 was shown to induce entire ectopic eyes [12], and several factors in combination could induce insulin-producing cells from exocrine pancreas cells [13]. These efforts culminated in the discovery that adult somatic cells can be reprogrammed to pluripotency and converted into distantly related cell types as diverse as lineages representing different germ layers [14, 15].

### The power of oocytes: installment of pluripotency

As briefly stated in the introduction, John Gurdon showed that even somatic cells can be reactivated by the oocyte to form mature fertile animals by transplanting nuclei of cultured intestinal cells of Xenopus tadpoles into enucleated oocytes (Fig. 1a) [3]. Subsequent experiments using different adult cell types only yielded swimming tadpoles, suggesting that the starting cell population may be restricted in their degree of plasticity or that reprogramming was not complete [16]. For many years, similar experiments in higher vertebrates failed and the field was dominated by the notion that mammalian cells have lost the plasticity of amphibian cells. It was only in 1996 when Ian Wilmut and his co-workers demonstrated that it is possible to generate live animals by nuclear transfer of adult mammalian cells when they successfully cloned the famous sheep Dolly [17]. In the coming years, many other mammalian species were successfully cloned including mice, rats, cats, dogs, cows, and others [18]. But for a long time, it remained unclear whether nuclear transfer could also reprogram human cells. Just a few years ago, this question was resolved and several groups convincingly showed that adult human fibroblasts can be transferred into human oocytes, which can give rise to blastocysts containing expandable pluripotent cells [19–21].

### The power of transcription factors: direct cell fate reprogramming

Developmental biology has focused on identification of transcription factors that are essential to induce cell type specific genetic programs; those factors are often expressed at distinct stages during differentiation to activate the desired genetic programs and are termed “selector genes.” One such example is the Drosophila *eyeless* gene (Pax6 in mammals) that is required for eye development [22]. Strikingly, Pax6 overexpression can induce the formation of eye structures in various appendages of the fly [12]. Similar effects have been observed using other selector genes, including the Hox family members *distalless* and *vestigial* (reviewed in [23]).

A different class are the so called “terminal selector genes” that regulate the identity of specific neuronal subtypes in *C. elegans* [24]. Terminal selector genes are transcription factors that are either alone or in combination specifically induced as the corresponding neuronal subtype is generated. Unlike classical selector genes, they stay expressed in these cells throughout the life of the animal and not only induce but also maintain subtype identity by activating key transcriptional modules necessary for the cell’s function and by repressing other terminal selector genes.

The basic helix-loop-helix (bHLH) transcription factor MyoD was the first factor identified that has the power to induce a cell lineage program in an unrelated cell type. Following a subtractive cDNA library screen, Harold Weintraub and colleagues cloned the cDNA coding for MyoD, which was sufficient to convert cultured mouse fibroblasts into beating muscle cells [11]. This work sparked the search for similar “master” lineage regulators for other cell types. By and large, however, this search was initially unsuccessful, and for many years, it was assumed that MyoD is unique.

Nevertheless, work in hematopoietic lineages continued to provide evidence for the existence of individual powerful lineage determination factors. Thomas Graf showed that the myeloid transcription factor C/EBPα is capable to directly convert B lymphocytes to macrophages in a stunningly efficient and rapid reprogramming process [25]. Another hematopoietic factor Pax5 was shown to maintain the B lymphocyte identity, and loss of function mutations led to transdifferentiation into other hematopoietic lineages [26]. Along similar lines, the eye-inducer Pax6 was also shown in a different cell context to convert neonatal astrocytes into neuronal cells [27].

All this work demonstrates that transcription factors are powerful, but their potency appeared somewhat limited, certainly as single factors. On the other hand, the successful nuclear transfer reprogramming experiments demonstrated that there must be specific reprogramming factors present in the oocyte that allow the installment of a pluripotent program. In 2006, Shinya Yamanaka and Kazutoshi Takahashi set out to screen for factors that could reprogram mouse fibroblasts into pluripotent cells. Establishing a reporter construct within the stem cell-specific Fbx15 locus allowed them to generate and isolate induced pluripotent stem (iPS) cells. Screening 24 candidate factors based on specific expression in pluripotent cells identified the now famous four reprogramming factors that can successfully convert fibroblasts to iPS cells: Oct4, Sox2, Klf4, and c-Myc [14]. A year later, the same group showed that the identical four factors also reprogram human cells (Fig. 1c) [28]. This finding was a game changer for the field and brought up the intriguing question what the limits of cell plasticity are.

Except for iPS cell reprogramming, all other successful transcription factor-based reprogramming examples reported up until that time were limited to conversions of closely related cell types. The question arose if also very distantly related cell types could be directly reprogrammed into each other. Tackling this problem, we attempted to convert mesoderm-derived fibroblasts into ectoderm-derived neural lineages. Assuming that neural reprogramming factors ought to be important lineage determination transcription factors that are also specifically expressed in neural cells, we chose over 20 candidate factors based on these criteria. From those candidates, we identified three factors, Brn2, Ascl1, and Myt1l, that in combination efficiently converted mouse and upon addition of Neurod1 also human fibroblasts into induced neuronal (iN) cells [15, 29]. The conversion efficiency of about 20% was surprisingly high, and the resulting iN cells had all principal biochemical, morphological, and functional properties of neurons. We subsequently showed that iN cells can also be derived from cells of definitive endodermal origin by converting terminally differentiated hepatocytes [30].

This work sparked great interest in the field and triggered several labs to further develop iN cell reprogramming techniques [31]. The successful generation of iN cells also inspired scientists to apply similar strategies to other cell lineages. To date, many important cell types can be generated through direct conversion from fibroblasts including cardiomyocytes, hepatocytes, intestinal cells, and blood progenitor cells (reviewed in [32]).

## Direct induction of progenitor cells

Often, strong lineage determination factors induce terminal differentiation. For example, MyoD induces mature skeletal muscle fibers skipping the proliferative myoblast stage, and also, the three reprogramming factors we found induce postmitotic neurons without a transient induction of neural precursor intermediate. For several applications, in particular for cell transplantation, the more plastic precursor cells would be desired because they are likely to better integrate into pre-existing host tissues than fully matured cells. Therefore, ensuing work has focused on the generation of precursor cell states from various different lineages.

We and others demonstrated that this can also be accomplished using transcription factor combinations unique for the desired progenitor cell population including oligodendrocyte precursor cells and tripotent neural progenitor cells [33–36]. However, an alternative approach was to transiently induce a pluripotent state using only brief exposure to iPS cell reprogramming factors such as Oct4, Sox2, Klf4, and c-Myc, without proper establishment of iPS cell lines, and rapidly followed by environmental differentiation cues. This approach has been successful to generate progenitor types of neural, hematopoietic, osteoblast, cardiac, and endothelial cells [37–42].

Two recent studies confirmed that this “indirect” reprogramming approach involves the transient induction of an authentic pluripotent state using elegant genetic lineage tracing techniques [43, 44].

## In vivo reprogramming

Another application of lineage reprogramming, potentially of clinical interest, is the in vivo reprogramming of endogenous cells to regenerate or replace damaged tissues (reviewed in [45]). Glial cells for example are the most abundant cells in adult brain and have progenitor-like features; therefore, they are perfectly suited to repair diseased or injured brains characterized by loss of neurons. Indeed, it has already been shown that glial cells such as astrocytes and oligodendrocyte precursor cells as well as perivascular cells can be converted into functional neuroblasts or neurons within the mouse brain and spinal cord [46–51]. Since induced neurons could also be converted from reactive glial cells in Alzheimer’s disease mouse models, this technology might be applicable for in vivo brain repair in the future [52].

Recent data suggest that it may even be possible to switch the identity of postmitotic neurons within the brain, because ectopic expression of the cortical transcription factor Fezf2 has been shown to reprogram upper layer neocortical neurons to lower layer 5 neurons [53, 54]. This conversion, however, was only successful in early postmitotic stages suggesting that neurons become less plastic as they mature. Another cell type successfully targeted for in vivo reprogramming is insulin-producing pancreatic β cells that can be converted from pancreatic exocrine cells in the adult mouse to decrease hyperglycemia caused by insulin deficiency observed in diabetes [13]. Even mouse intestinal and liver cells could recently be reprogrammed to insulin-secreting cells, suggesting potential therapeutic value for diabetic patients by in vivo reprogramming of non-pancreatic cell types [55, 56]. In addition, it is possible to generate hepatocyte-like cells from myofibroblasts that could reduce early signs of chemical and cholestasis-induced liver fibrosis in the mouse [57]. Besides those cell types, cardiomyocytes are an attractive target for regenerative reprogramming. Indeed, induced cardiomyocyte-like cells could be efficiently generated through in vivo reprogramming from endogenous cardiac fibroblasts and enhanced cardiac function after heart injury in mice [58, 59]. However, cardiomyocytes generated by reprogramming exhibit phenotypic and electrophysiological heterogeneity causing a potential risk of arrhythmias [60]. Like for β cells, induction of cardiomyocytes is much more efficient in vivo then in vitro, highlighting the importance of the in vivo niche for the reprogramming process [58, 59, 61].

Sensory receptor cells that reside in the retina, olfactory epithelium, and inner ear are also clinically relevant cell types for potential therapeutic reprogramming. Along these lines, Ascl1 has been shown to convert retinal Müller glia to neuronal fate in injured mice [62]. Importantly, young mice responded more efficiently to Ascl1 overexpression than older mice [62], suggesting that age-associated changes restrict reprogramming as recently observed in vitro [63]. The sequence-related bHLH factor Atoh1 (Math1) was also successfully used for induction of hair cells in mouse and rat inner ear, another important sensory cell type [64, 65].

### Mechanism, mediators, and roadblocks of reprogramming

**Fig. 2:**
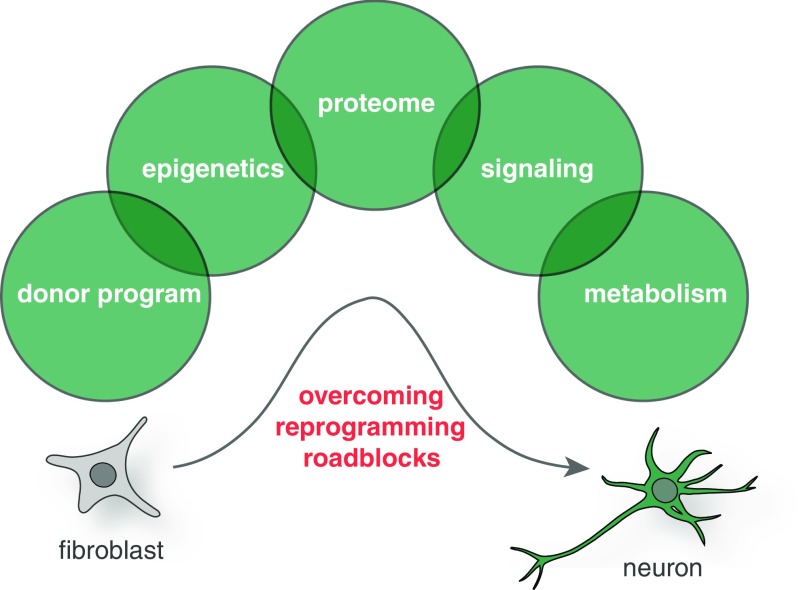
Cell fate roadblocks for lineage reprogramming. Cell identity is a function of many parameters including gene expression, epigenetic configuration, protein composition, signaling pathway activity, and metabolism. Several studies suggest that modulation of any of these parameters not only follows but also dictates physiological and induced cell fate changes. It will therefore be essential to devise methods to monitor and potentially modulate all these parameters in order to faithfully generate cells for disease modeling and regenerative medicine

Understanding the mechanism and identifying the key roadblocks and mediators that hinder and enable cell fate changes, respectively, will be an essential task for the field in order to ultimately orchestrate the reprogramming process in a tightly controlled manner required for disease modeling and regenerative medicine. Below, we will discuss some of the key aspects of the reprogramming mechanism, and we will consider some principal obstacles cells are facing when induced to change fate (Fig. 2).

## Pioneer transcription factors

The DNA binding of most transcription factors is highly dependent on the chromatin configuration in a given cell. There is ample evidence for the notion that the chromatin modifies the binding affinity of ordinary transcription factors in addition to the affinity based on DNA sequence. One of the most obvious barriers for transcription factor access are the multi-unit nucleosomes that have high affinity for DNA and bind about 146 bp of linear DNA sequence in about 1.7 superhelical turns. As so often in biology, there are exceptions from the rule. A small handful of transcription factors seem to behave differently and have been found to directly access nucleosomal DNA (or “closed chromatin”). This class of transcription factors has been termed “pioneer” factors. For instance, FoxA and GATA factors were shown to initiate liver and heart development in a pioneering mechanism (reviewed in [66]).

It turns out that many of the reprogramming factors, identified in independent functional screens, belong in fact into this “pioneer” category [67–69]. Three of the four iPS cell reprogramming factors, namely Oct4, Sox2, and Klf4 (OSK), but not c-Myc have been shown to possess pioneer factor activity [67, 68]. More recent work has shown that despite their pioneering potential, the binding of these three factors is still highly context dependent illustrated by the observation that their binding varies along different reprogramming stages and also changes when other reprogramming factors are co-expressed in fibroblasts [70]. Thus, despite their pioneering capability, these iPS cell reprogramming factors are still binding in a chromatin-dependent manner.

Ascl1, one of the key reprogramming factors to generate iN cells from fibroblasts, seems to behave in a fundamentally different way. Its binding pattern in fibroblasts immediately after overexpression and its binding pattern in normal neural precursor cells is in fact very similar, even though these two cell types exhibit a completely distinct chromatin state [69]. Ascl1, therefore, appears to have additional qualities compared to other pioneer factors, in the sense that it actually binds its neuronal targets seemingly independent of the chromatin state of the cells. We called this property “on target” pioneer factor activity.

Finally, the main reprogramming factor that converts B lymphocytes to macrophages, C/EBPα, has recently been reported to also act as pioneer factor during reprogramming [71].

In summary, pioneer factor activity seems to be a common property of reprogramming factors. Since they engage with silent chromatin and can at least in part override chromatin barriers, their expression must be tightly regulated during normal development, to ensure proper lineage specification.

## Donor program repression

Much attention is being devoted to the induction of target cell programs as cells differentiate or are reprogrammed. However, equally important is the downregulation of the donor cell program and the silencing of undesired transcriptional programs during new lineage acquisition [72]. In some cases, continued expression of exogenous reprogramming factors is required to maintain the newly acquired cell state, and downregulation of the factors leads to their reversion towards the donor fibroblast identity [73]. Failure to silence the expression programs of the initial cell population and induction of unwanted programs might explain immature phenotypes observed upon reprogramming [74, 75]. Most reprogramming regimes seem to repress the donor cell specific program before induction of the target program such as in the reprogramming of fibroblasts to iPS cells [76], pre-B cells to macrophages [77], fibroblasts, and hepatocytes to neurons [30, 69, 72]. The addition of three mature hepatocyte-enriched transcription factors, C/EBPα, ATF5, and PROX1, in combination with hepatic reprogramming factors HNF1A, HNF4A, and HNF6 resulted in the induction of human-induced hepatocytes, suggesting that specific factors might contribute to donor program silencing [78].

The understanding of how the donor cell program is silenced in these induced cell conversions is only in its infancy. A recent study on iPS cell reprogramming suggested that the reprogramming factors themselves initially bind and decommission fibroblast enhancers and gradually activate pluripotency enhancers [70]. Investigating the iN cell reprogramming mechanism, we found that Myt1l, one of the three reprogramming factors, appears to be dedicated to suppress the fibroblast and many other non-neuronal programs whereas activation of the neuronal program is accomplished by the “on target pioneer” factor Ascl1 [69, 79]. Therefore, repressing alternative cell fates along with concomitant induction of cell type specific programs enable faithful and efficient binary decisions during cell fate reprogramming.

## Epigenetic regulators

Cell identity is largely driven by the overall gene expression, which in turn is regulated by the chromatin state. It therefore seems likely that also epigenetic mechanisms constitute important barriers for reprogramming (reviewed in [80]). In fibroblasts, genes required to establish pluripotency were shown to be “locked” initially within H3K9me3-enriched heterochromatin domains that restrict the access of the reprogramming factors [67]. Accordingly, reducing H3K9me3 levels by knockdown of the histone methyltransferases SUV39H1/H2 lowered this barrier to reprogramming [67]. Inversely, a “trivalent” chromatin signature consistent of H3K4me1, H3K27ac, and H3K9me3 was enriched at the binding sites of the pioneer factor Ascl1 in fibroblasts. Its cell type specific presence is predictive of the reprogramming outcome and “erasure” of H3K9me3 by overexpression of the histone demethylase KDM4D impaired neuronal reprogramming, further suggesting that epigenetic barriers are essential for cell fate conversion [69].

Direct methylation of DNA is considered another robust epigenetic mechanism stabilizing cell lineage programs. Its global depletion by treatment with the drug 5-azacytidine relieves this break, inducing the differentiation of fibroblasts into several lineages including muscle cells, adipocytes, and chondrocytes [81]. New approaches to specifically rewrite the epigenome in a sequence-specific manner might allow directed reprogramming of cells that are blocked by epigenetic barriers [82, 83].

In addition, there are multiple examples that transcriptional regulators work in conjunction with chromatin-modifying factors. In Pax6-mediated reprogramming of mouse glia to neurons, it was shown that the chromatin remodeling complex member Brg1 (also known as Smarca4) is required for this process [84]. Compatible with this insight, the formation of iN cells from human fibroblasts with a combination of transcription factors and the microRNAs miR9/9* and 124 involved the accurate induction and assembly of the neuronal-specific Brg1-associated factor (BAF) complex, also known as SWI/SNF complex [85, 86]. Some of the microRNAs used in this reprogramming protocol block expression of the chromatin complex REST, which is a specific repressor of neuronal genes and therefore needs to be silenced in neurons. Another target of miR124 is a protein called PTB, which in turn was shown to regulate iN cell reprogramming and it was proposed that just reducing PTB levels promotes iN cell formation from fibroblasts [87, 88]. Since PTB blocks miRNA-mediated activity of the REST complex, its depletion enables expression of multiple miRNA-regulated neuronal genes.

An early study showed that the combination of transcription and chromatin factors enables the reprogramming of non-cardiac fetal cells into cardiomyocytes [89]. This study further showed that BAF60c, a cardiac specific subunit of the BAF complex, enabled the binding of Gata4 to cardiac-specific genes. Moreover, depletion of the polycomb complex member Bmi1 appeared to de-repress cardiac genes and enhance reprogramming to cardiomyocytes [90].

Finally, the histone chaperone complex CAF-1 has recently been shown to limit reprogramming towards several cell types, including iPS cells and neurons [91]. Together, these studies indicate that chromatin factors and transcriptional regulators are highly dependent on each other and work together to accomplish the remodeling of the chromatin that in turn dictates lineage identity.

### Future perspectives and biomedical applications

Our experimental command on lineage reprogramming, discovered by basic researchers driven by their scientific curiosity, has transformed biomedical research over the last few years. Rather than being studied in a handful of laboratories, today every major academic institution and pharmaceutical company entertains stem cell facilities that serve their scientists to provide human cell types for research. Lineage reprogramming has become a new asset in the arsenal of research with the goal to investigate pathomechanisms and develop therapeutic approaches for various human diseases.

There are four main areas where lineage reprogramming and pluripotent stem cells are or could be applied to enhance biomedical research (Fig. 3):

1. Disease modeling: It is now possible to obtain skin or blood cells from patients and convert them into essentially any other desired cell type relevant for the particular underlying disease. This new kind of disease modeling has the great advantage that actual human patient cells are used rather than cell line or animal models, which might not always reflect the complexity of human-specific traits including the mechanisms of human diseases. This application is perhaps the one with the highest impact of lineage reprogramming on biomedical research. With all excitement about this new way to study diseases, it is also clear that there are currently obvious limitations. Cultured cells are not comparable to three-dimensional organs of the body and only minimally reflect the complex interaction of multiple different cell types. More sophisticated models will be needed combining tissue engineering with reprogramming and stem cell approaches. Efforts are on the way to manufacture “organs on a chip” to mimic at least some aspects of physiological organ interactions [92]. An intriguing alternative approach is three-dimensional differentiation as so called “organoids.” Pluripotent stem cells have the remarkable property to self-organize, thus imitating early embryonic structures that can be exploited to generate at least embryonic or fetal embryoid tissue structures [93].

**Fig. 3:**
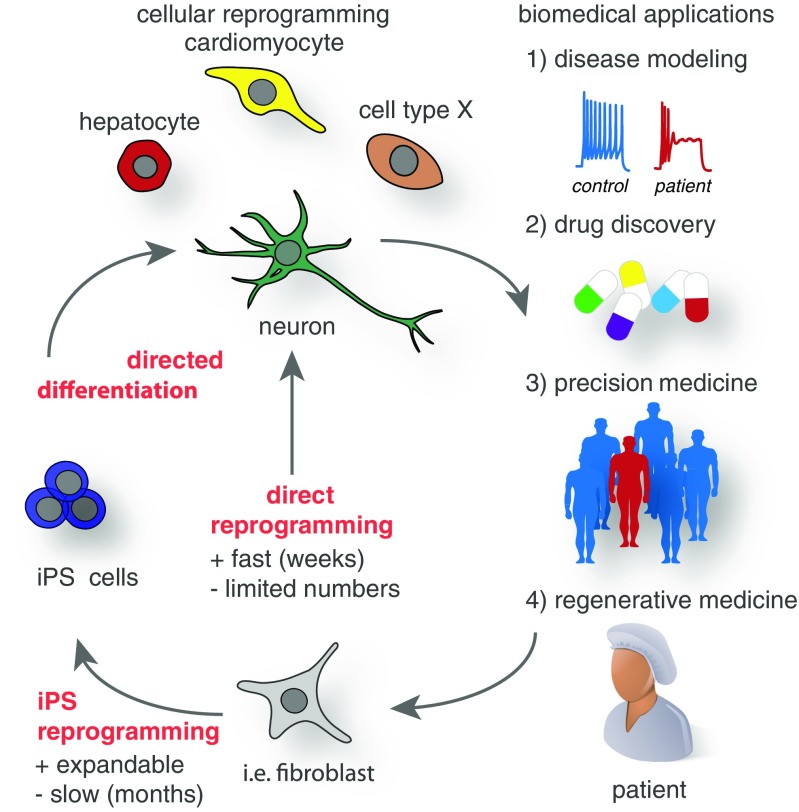
Current cellular reprogramming technologies and future biomedical applications. Cell fate changes can be induced to generate human cells for (1) in vitro disease modeling and (2) drug discovery as well as for potential (3) personalized drug screens in precision medicine and (4) regenerative applications in the near future. Donor cells such as fibroblasts can be directly reprogrammed to many cell types of biomedical interest, while this process usually is very fast it often only generates a limited amount of cells. Alternatively, donor cells can first be reprogrammed to induced pluripotency and subsequently directed to the intended fate, in general this procedure is more time consuming but in theory generates unlimited amounts of cells for biomedical applications
2. Drug discovery: Reprogramming could be used to generate specific cell types from a large cohort of patients representing various ethnicities and genetic backgrounds. Newly developed drugs could be evaluated in such cells as a “clinical trial in a dish” before the drugs would be tested in people. This approach could be used for both general efficacy and side effect evaluation. Given the often extensive financial burden of clinical trials, such intermediate in vitro assays would be of high interest to the pharmaceutical industry.
3. Precision medicine: Many already approved drugs have deleterious side effects like heart arrhythmias in a small and unpredictable subpopulation of patients. Reprogramming methods could facilitate precision medicine by testing drugs with known serious side effects first on reprogrammed patient cells before administration. For instance, potential arrhythmic-inducing drugs could be first tested on reprogrammed cardiomyocytes derived from individual patients. Assuming that such reprogrammed cardiomyocytes express the full panel of ion channels and pumps present in the heart, it would seem in principle straightforward to identify drugs that bind and alter the function of these channels in individual patients that may carry unknown genetic variants predisposing them to develop side effects. Given the complexity of the genome, such variants are very difficult to predict and identify. The advantage of the reprogramming approach outlined here is that no prior knowledge on risk factors is needed, since the tested cardiomyocytes will be derived from reprogrammed patient cells that carry the identical genetic background in a personalized manner.
4. Regenerative medicine: Finally, but not least, reprogramming could be used in novel regenerative medicine approaches. Principally, any cell type could be manufactured from easily accessible tissue such as skin or blood. Therefore, current cell replacement therapies such as envisioned for neurodegenerative diseases could be performed using autologous cells, which would eliminate the complication of an immune rejection of the graft. In cases where immune rejection is of major concern, an autologous source could justify the higher development and manufacturing costs that are associated with a more involved manufacturing procedure using cellular reprogramming. Importantly, reprogramming could be combined with gene editing, thereby allowing new therapeutic approaches for rare monogenetic diseases, but also the genetic engineering of autologous cells to deliver therapeutic factors to otherwise inaccessible structures. An intriguing shortcut for cell replacement could be an in vivo reprogramming approach, discussed above. Rather than reprogramming cells ex vivo followed by cell transplantation, therapeutic reprogramming could be accomplished in vivo by direct delivery of reprogramming vectors to the target organs.
